# MicroRNAs Involved in Regulatory Cytoplasmic Male Sterility by Analysis RNA-seq and Small RNA-seq in Soybean

**DOI:** 10.3389/fgene.2021.654146

**Published:** 2021-05-12

**Authors:** Chunbao Zhang, Fuyou Fu, Chunjing Lin, Xiaoyang Ding, Jingyong Zhang, Hao Yan, Pengnian Wang, Wei Zhang, Bao Peng, Limei Zhao

**Affiliations:** ^1^Soybean Research Institute, The National Engineering Research Center for Soybean, Jilin Academy of Agricultural Sciences, Changchun, China; ^2^Saskatoon Research Centre, Agriculture and Agri-Food Canada, Saskatoon, SK, Canada

**Keywords:** microRNA, degradome, RNA-seq, cytoplasmic male sterility, soybean

## Abstract

Cytoplasmic male sterility (CMS) is an important plant characteristic for exploiting heterosis to enhance crop traits during breeding. However, the CMS regulatory network remains unclear in plants, even though researchers have attempted to isolate genes associated with CMS. In this study, we performed high-throughput sequencing and degradome analyses to identify microRNAs (miRNAs) and their targets in a soybean CMS line (JLCMS9A) and its maintainer line (JLCMS9B). Additionally, the differentially expressed genes during reproductive development were identified using RNA-seq data. A total of 280 miRNAs matched soybean miRNA sequences in miRBase, including mature miRNAs and pre-miRNAs. Of the 280 miRNAs, 30, 23, and 21 belonged to the miR166, miR156, and miR171 families, respectively. Moreover, 410 novel low-abundant miRNAs were identified in the JLCMS9A and JLCMS9B flower buds. Furthermore, 303 and 462 target genes unique to JLCMS9A and JLCMS9B, respectively, as well as 782 common targets were predicted based on the degradome analysis. Target genes differentially expressed between the CMS line and the maintainer line were revealed by an RNA-seq analysis. Moreover, all target genes were annotated with diverse functions related to biological processes, cellular components, and molecular functions, including transcriptional regulation, the nucleus, meristem maintenance, meristem initiation, cell differentiation, auxin-activated signaling, plant ovule development, and anther development. Finally, a network was built based on the interactions. Analyses of the miRNA, degradome, and transcriptome datasets generated in this study provided a comprehensive overview of the reproductive development of a CMS soybean line. The data presented herein represent useful information for soybean hybrid breeding. Furthermore, the study results indicate that miRNAs might contribute to the soybean CMS regulatory network by modulating the expression of CMS-related genes. These findings lay the foundation for future studies on the molecular mechanisms underlying soybean CMS.

## Introduction

Soybean is an important crop that is cultivated for its protein and oil content. In some countries, especially China, soybean production is less profitable for farmers than the production of corn, rice, or other crops, resulting in yearly decreases in the arable land area used for cultivating soybean. Crop yields may be increased by exploiting heterosis (Ivanov and Dymshits, 2007; Virmani, 2012). One of the most important methods for exploiting heterosis involves the application of cytoplasmic male sterility (CMS). Specifically, CMS has been broadly used for breeding major crop species (Yuan, 1966; Bosacchi et al., 2015). Heterosis has been exploited to improve specific soybean traits (Zhao et al., 2004). In 1995, Ru Nan Tian E Dan male sterile cytoplasm was developed as the first soybean CMS system (i.e., RN-CMS; Sun et al., 2001). In 2002, HybSoy 1 became the first soybean hybrid bred using the RN–CMS system, with an average yield of more than 20% greater than that of the control line (Zhao et al., 2004). By 2020, 23 soybean hybrids had been bred with the RN–CMS system. However, it remains unclear how RN–CMS works.

The small RNAs (sRNAs) are short [approximately 18–30 nucleotides (nt)] non-coding RNA molecules that can regulate gene expression in the cytoplasm and nucleus via post-transcriptional gene silencing, chromatin-dependent gene silencing, or RNA activation. The three classes of sRNAs are microRNAs (miRNAs), small interfering RNAs, and Piwi-interacting RNAs. The miRNAs are a class of short (20–24 nt) non-coding RNAs that regulate gene expression at the post-transcriptional level by degrading their target mRNAs and/or inhibiting translation (Banisch et al., 2012).

Previous research confirmed that sRNAs, including miRNAs, can modulate anther and pollen development, leading to male sterility (Chambers and Shuai, 2009; Grant-Downton et al., 2009; Wang et al., 2009; Xing et al., 2010; Shen et al., 2011; Wei et al., 2012; Yang J. et al., 2013). For example, miR156, miR167, and miR399 influence pollen development in *Arabidopsis thaliana* and citrus plants (Wu et al., 2006; Wang et al., 2009, 2020; Xing et al., 2010; Wei et al., 2012). An earlier deep-sequencing analysis proved that miRNAs contribute to the flower and pollen development of a soybean CMS line and its maintainer line (Ding et al., 2016). Recent studies revealed the opposite expression patterns of gma-miR156b and its target *GmSPL* genes, including *GmSPL9*, in flowers during the early flower bud development stage of the soybean CMS A-line and its maintainer B-line (Ding et al., 2019, 2020). In this study, RN-CMS line JLCMS9A and its maintainer line JLCMS9B were analyzed by sequencing their sRNAs to identify which sRNAs, especially miRNAs, induce sterility.

## Results

### sRNA Sequencing and Degradome Profiling of JLCMS9A and JLCMS9B

**FIGURE 1:**
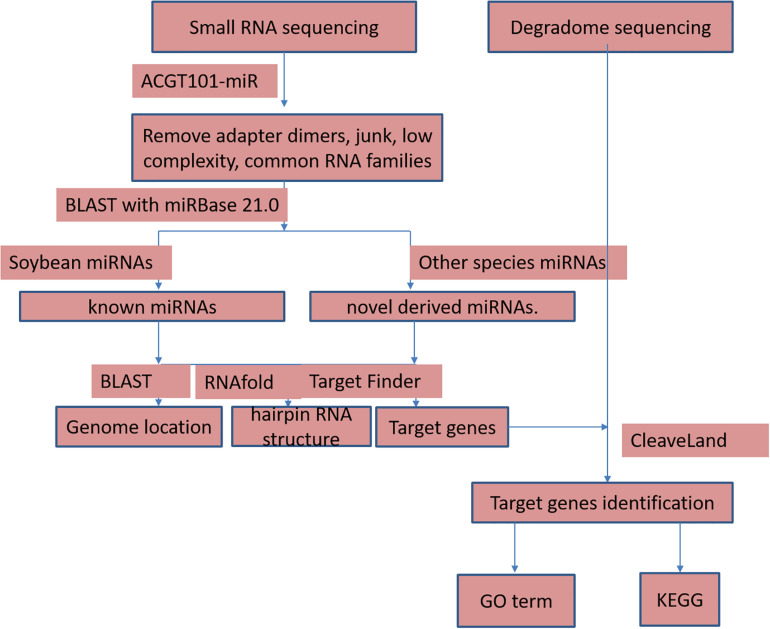
The developmental bioinformatics pipeline of small RNA sequencing analysis in this study.

**FIGURE 2:**
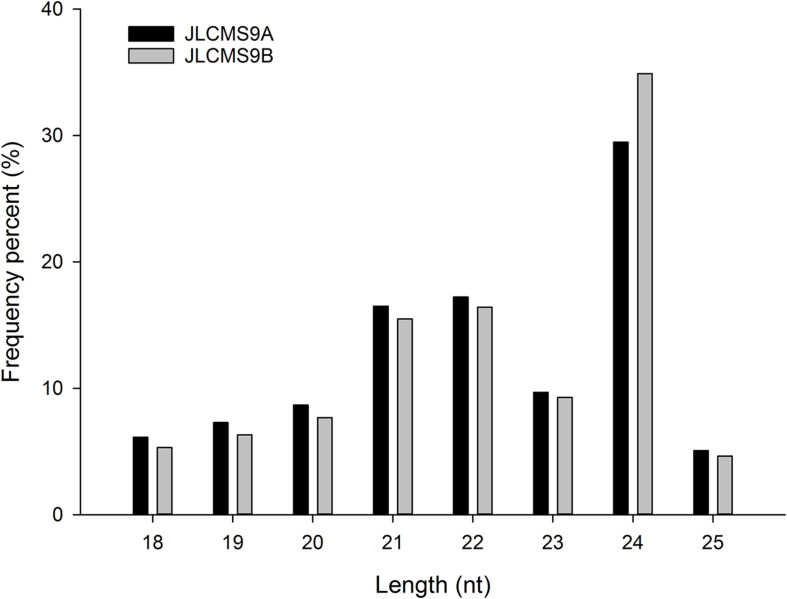
The length distribution of small RNAs in JLCMS9A and JLCMS9B flower buds.

The high-throughput sequencing of two independent sRNA libraries for the flower buds of the CMS line JLCMS9A and its maintainer line JLCMS9B generated 28,736,898 and 32,977,823 raw reads, respectively (Supplementary Table 1). Our small RNA bioinformatics analysis was performed using the development pipeline (Figure 1). After removing adapter contaminants, oversized insertions, low-quality reads, poly-A tags, short and long tags (tags < 18 nt and >25 nt), and other non-coding RNAs (rRNA, snoRNA, snRNA, and tRNA), 5,996,889 and 7,397,799 unique clean reads (18–25 nt long), including 3,639,335 and 4,791,157 valid unique sRNA reads, were obtained for JLCMS9A and JLCMS9B, respectively, (Supplementary Table 1). Most of the sRNAs in the two libraries were 21–24 nt long, with 24 nt being the most common length, followed by 21 and 22 nt (Figure 2). This is consistent with previously reported sRNA length distributions for various soybean tissues, including the roots, nodules, flowers, developing seeds, and cotyledons (Joshi et al., 2010; Song et al., 2011; Goette et al., 2014).

### Identification of Known miRNAs

All sRNA sequences were mapped to known soybean miRNAs in the miRBase 21.0 database^1^ to identify conserved miRNAs. In the present study, 1,327 miRNAs from 250 families were predicted and corresponded to 511 pre-miRNAs (Supplementary Table 2). Additionally, 21 nt was the most common length among the known miRNAs, followed by 22 and 24 nt (Supplementary Figure 1). Of the identified miRNA families, miR166 had the most members (Hu et al., 2016), followed by miR171 and miR169, with 48 and 45 members, respectively (Supplementary Figure 2). In contrast, most families had fewer than five members, with 73, 98, 13, and 19 families comprising one, two, three, and four members, respectively, (Supplementary Table 3). Among the 250 miRNA families, 39 identified families were conserved in other species (47 species in the miRBase 21.0 database). The rest were non-conserved soybean-specific miRNA families, including 13, 8, and 15 that were revealed to be conserved at high (>10 plant species), moderate (five to nine plant species), and low (two to four plant species) levels on the basis of a previous classification (Zhang et al., 2006). Additionally, there were 183 non-conserved miRNA families (Supplementary Table 4).

### Identification of Known Conserved miRNAs

To assess the accuracy of the miRNA predications, the miRNAs were divided into six groups. A total of 828 conserved miRNAs (820 and eight in gp1a and gp1b, respectively) and 491 non-conserved miRNAs (71, 238, 20, and 162 in gp2a, gp2b, gp3, and gp4, respectively) were identified (Supplementary Figure 3 and Supplementary Table 2). In this study, only conserved miRNAs were analyzed further. A total of 280 miRNAs matched soybean miRNA sequences in the miRbase database, including mature miRNA and pre-miRNA sequences. Moreover, 85 and 170 miRNAs were highly and moderately expressed in the JLCMS9A and JLCMS9B flower buds, respectively. Among the 280 miRNAs, 30, 23, and 21 belonged to the miR166, miR156, and miR171 families, respectively. Accordingly, known conserved miRNAs appear to regulate the flower bud development of JLCMS9A and JLCMS9B.

### Identification of Known Newly Conserved miRNAs

Known newly conserved miRNAs usually match confirmed miRNA sequences in miRBase, but we detected some sequence diversity in this study. For example, the gma-miR156b_L + 1R-1 sequence (TTGACAGAAGAGAGAGAGCAC) was confirmed using the miRBase database, but it differed from the gma-miR156b sequence (TGACAGAAGAGAGAGAGCACA; Supplementary Table 2). A total of 144 known newly conserved miRNAs were confirmed in this study. Additionally, 15 and 92 miRNAs were, respectively, identified as highly and moderately expressed in the JLCMS9A and JLCMS9B flower buds. Among these 144 miRNAs, 10, eight, and five were identified as members of the miR393, miR395, and miR169 families, respectively.

### Identification of Novel miRNAs

Novel miRNAs, especially new 5p and 3p sequences, are not included in miRBase. In this study, 410 novel miRNAs were identified, including gma-miR156m-p3, which was a new 3p sequence (Supplementary Table 2). A total of 404 novel miRNAs were identified in this study. Among the 144 miRNAs, 10, eight, and five miRNAs belonged to the miR393, miR395, and miR169 families, respectively. However, only the following six were highly expressed in soybean flower buds: gma-miR166e-p5, gma-miR482c-p5, gma-miR1513c-p5, gma-miR4997-p3, gma-miR5368-p5, and gma-miR5368-p3_2ss1AT18CA. Among the 404 novel miRNAs, 26, 15, and 15 miRNAs belonged to the miR1520, miR156, and miR171 families, respectively. Thus, the novel miRNAs were not highly abundant in the JLCMS9A and JLCMS9B flower buds.

### Identification of miRNA Target Genes via a Degradome Analysis

Independent degradome libraries for the JLCMS9A and JLCMS9B flower buds were constructed and sequenced, resulting in 13,604,916 and 15,876,060 raw reads, respectively (Supplementary Table 5). After eliminating the adapter sequences and/or low-quality reads from the raw reads longer than 15 nt, 13,484,912 (99.12%) and 15,741,865 (99.14%) were perfectly aligned to the soybean reference genome (Gmax_275_v2.0; Supplementary Table 3). After CleaveLand4 was used to process and analyze the data (Addo-Quaye et al., 2009), 1,617 target genes were predicted to be cleaved by 349 miRNAs in JLCMS9A and JLCMS9B.

**FIGURE 3:**
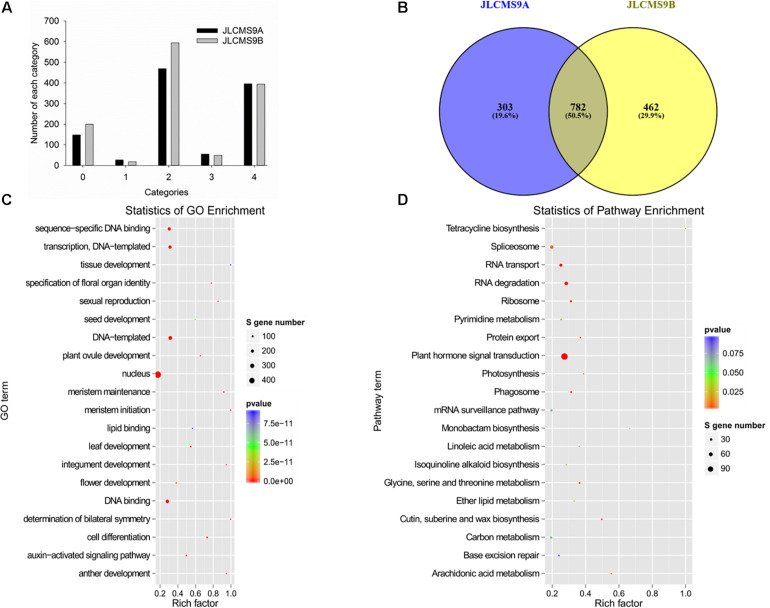
The profiles of small RNAs (sRNAs) and target genes in JLCMS9A and JLCMS9B. **(A)** Number of sRNAs in different categories. **(B)** Number of unique and common sRNAs in JLCMS9A and JLCMS9B. **(C)** Gene Ontology (GO) functional analysis of target genes. **(D)** Kyoto Encyclopedia of Genes and Genomes (KEGG) pathway analysis of target genes.

The sliced-target genes were divided into five categories (0, 1, 2, 3, and 4) on the basis of the relative abundance of tags at the target sites as previously described (Addo-Quaye et al., 2008; Li et al., 2010; Liu et al., 2014). The miRNAs and their targets in the five categories are presented in Supplementary Table 6 and Figure 3A. More gene targets were detected in JLCMS9B (1,095) than in JLCMS9A (1,255). Specifically, the number of gene targets in categories 0 and 2 was substantially higher for JLCMS9B than for JLCMS9A (Figure 3A). A total of 303 and 462 unique targets were, respectively, predicted for JLCMS9A and JLCMS9B, in addition to 782 common targets following the degradome analysis (Figure 3B).

The target gene analysis revealed that a single miRNA can simultaneously regulate the expression of several target genes, usually from a large gene family. The predicted highly conserved miRNAs, such as miR169, miR156, miR396, miR166, miR172, and miR171 family members, regulated multiple target genes. For example, the miR167 family members were detected as regulators of the expression of 206 target genes, including those encoding mitochondrial carrier proteins, small nuclear ribonucleoprotein component-like proteins, MYB domain proteins, transcription initiation factors, and squamosa promoter-binding-like (SPL) proteins (Supplementary Table 6). Moreover, a single mRNA may be targeted by multiple miRNAs, even from different miRNA families. For example, *SPL6* was identified as a target for 15 and three miRNAs from the miR156 and miR157 families, respectively (Supplementary Table 6). Meanwhile, the expression of six selected miRNAs were tested using quantitative real-time PCR (qRT-PCR) analysis, which further validated the differential expression data obtained from sequencing on this study. The expression pattern of selected miRNAs was consistent with the sequence reads (Supplementary Figure 4).

### GO and KEGG Analysis of Target Genes

**FIGURE 4:**
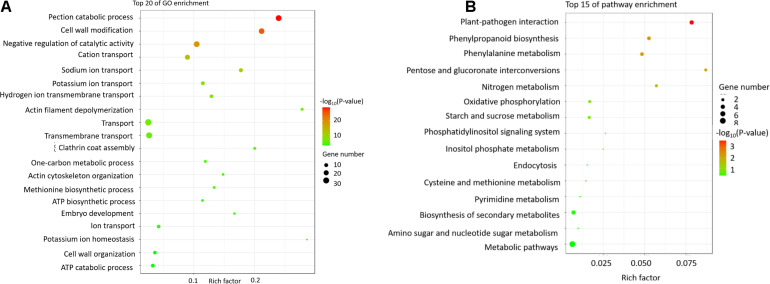
The GO and KEGG pathway analyses of the differentially expressed genes between JLCMS9A and JLCMS9B. **(A)** Top 20 GO terms. The *x*-axis presents the GO terms, whereas the *y*-axis presents the enrichment factors. **(B)** Top 15 KEGG pathways. The *x*-axis presents the KEGG pathways, whereas the *y*-axis presents the enrichment factors.

The identified miRNA target genes were subjected to a Gene Ontology (GO) analysis (Figure 3C) and assigned to metabolic pathways in the Kyoto Encyclopedia of Genes and Genomes (KEGG) database (Figure 3D) to functionally characterize the target genes and elucidate the regulatory effects of miRNAs on pollen development. The GO analysis indicated that the target genes affected various biological processes, cellular components, and molecular functions, including transcriptional regulation, the nucleus, meristem maintenance, meristem initiation, cell differentiation, auxin-activated signaling, plant ovule development, and anther development (Figure 4A). The enriched KEGG pathways among the target genes were associated with the spliceosome, RNA transport, RNA degradation, the ribosome, and plant hormone signal transduction. These results may reflect the importance of these miRNAs for regulating gene expression during pollen development in soybean plants.

### Identification of Differentially Expressed Genes

A total of 103,976,346 and 90,354,809 high-quality RNA-seq reads were generated for JLCMS9A and JLCMS9B, respectively, using a Life Technologies Ion Proton sequencer (Supplementary Table 2). After filtering, trimming the adapters, and removing the low-quality reads, 85,715,721 and 75,773,490 high-quality reads were retained for JLCMS9A and JLCMS9B, respectively. These reads were mapped to the soybean reference genome (Gmax_275_v2.0) using HISAT2, with an average matching rate of 86 and 84% for JLCMS9A and JLCMS9B, respectively (Supplementary Table 2; Kim et al., 2015).

The DEGs were detected following the pair-wise comparisons of the two lines using the DEGseq algorithm, with a false discovery rate ≤ 0.05 and [log_2_(fold-change)] ≥ 1 applied as the threshold (Wang et al., 2010). A total of 440 DEGs were identified between JLCMS9A and JLCMS9B (Supplementary Table 4). The expression levels of 428 and 12 of these DEGs were down-regulated and up-regulated, respectively, in JLCMS9A relative to the corresponding expression level in JLCMS9B. Interestingly, 174 of the down-regulated DEGs were only expressed in JLCMS9B, and five of the up-regulated DEGs were uniquely expressed in JLCMS9A. Thus, CMS in soybean may be related to the lack of expression and the induced expression of these 174 and 5 genes, respectively. These DEGs may be closely related to soybean CMS.

### Gene Ontology Annotation and Kyoto Encyclopedia of Genes and Genomes Pathway Enrichment Analysis

On the basis of the annotated GO terms, 440 DEGs were assigned to 25 categories (*P* < 10^–5^), including 10 biological processes, four cellular components, and 11 molecular function categories (Figure 4A). Among the biological process categories, pectin catabolic process was the main annotated GO term, followed by cell wall modification and catalytic activity. Among the cellular component categories, cell wall was the most significantly enriched GO term. Of the molecular function categories, the most common GO term among the DEGs was pectinesterase activity, followed by aspartyl esterase activity and enzyme inhibitor activity.

The enriched metabolic pathways among the DEGs were identified with the KEGG pathway database. Specifically, 37 DEGs were assigned to 15 KEGG pathways (Figure 4B), including biosynthesis of secondary metabolites, phenylalanine metabolism, and phenylpropanoid biosynthesis.

### miRNA-Gene Regulatory Network Analysis

**FIGURE 5:**
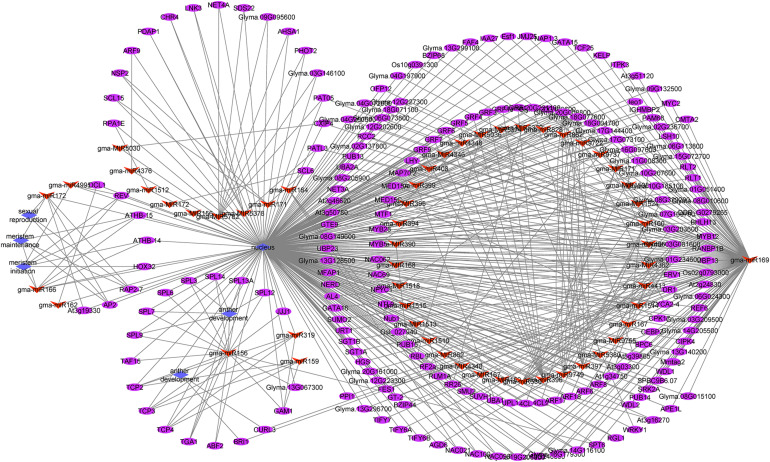
The regulatory network comprising GO terms, microRNAs (miRNAs), and target genes in JLCMS9A and JLCMS9B. Diamonds, ellipses, and arrowheads represent the GO terms, target genes, and miRNAs, respectively.

**FIGURE 6:**
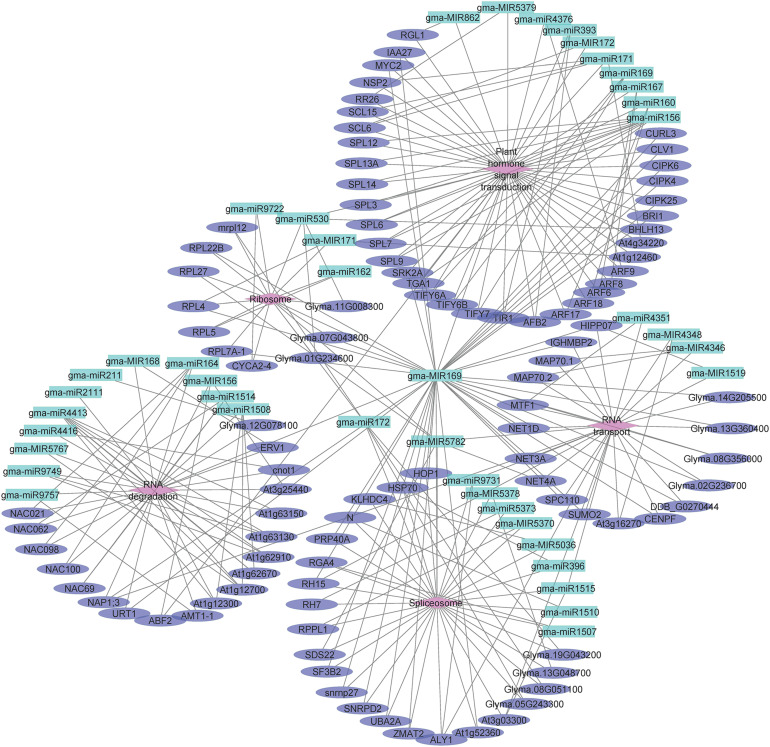
The regulatory network comprising KEGG pathways, microRNAs (miRNAs), and target genes in JLCMS9A and JLCMS9B. Diamonds, ellipses, and arrowheads represent the KEGG pathways, target genes, and miRNAs, respectively.

To investigate the functions of differentially expressed miRNAs and miRNA target genes, a regulatory network was built for the miRNAs and target genes on the basis of the enriched GO terms and KEGG pathways. Figure 5 presents the regulatory network for 204 target genes, 55 miRNAs, and five GO terms (sexual reproduction, anther development, meristem maintenance, meristem initiation, and nucleus). Figure 6 presents the regulatory network for 103 target genes, 42 miRNAs, and five KEGG pathways (ribosome, RNA degradation, RNA transport, spliceosome, and plant hormone signal transduction). The members of the gma-miR169 family were the most common regulators of the miRNA target genes.

## Discussion

### Identification of miRNAs in JLCMS9A and JLCMS9B Flower Buds

In plants, sRNAs are pivotal regulators of male fertility during anther and pollen development (Chambers and Shuai, 2009; Grant-Downton et al., 2009; Wang et al., 2009; Xing et al., 2010; Shen et al., 2011; Wei et al., 2012; Yang J. et al., 2013). A previous deep-sequencing study revealed the involvement of miRNAs in the flower and pollen development of a soybean CMS line and its maintainer line (Ding et al., 2016). However, the mechanism underlying the relationships among the miRNAs, target genes, and pollen development remained unclear in soybean. In the present study, to clarify the regulation of miRNAs and target genes during pollen development in soybean, CMS line JLCMS9A and its maintainer line JLCMS9B were analyzed by sRNA, RNA, and degradome sequencing. The analyzed sRNAs were mainly 24 nt long, but sRNAs that were 21 and 22 nt long were also relatively common (Figure 2). The sRNA lengths were consistent with the results of earlier related investigations of other plants, including *A. thaliana* (Rajagopalan et al., 2006), *Citrus sativus* (Kou et al., 2012; Fang et al., 2016), *Medicago truncatula* (Wang et al., 2011; Chen et al., 2012), *Oryza sativa* (Wei et al., 2011), and *Zea mays* (Li et al., 2013).

Several researchers identified miRNAs in diverse male sterile crops, including maize (Shen et al., 2011), rice (Zhou et al., 2012), cotton (Wei et al., 2013b; Yang X. et al., 2013; Yu et al., 2020), wheat (Bai et al., 2017; Sun et al., 2018), and *Brassica juncea* (Yang J. et al., 2013). In the current study, miRNAs and their target genes were identified following the deep-sequencing analysis of the flower buds from soybean CMS and maintainer lines. Additionally, the DEGs between the CMS and maintainer lines were identified. The 828 conserved, 491 non-conserved, and 410 novel miRNAs revealed in this study exceeded the corresponding number of miRNAs in an earlier study (Ding et al., 2016). The known miRNAs have been classified into four categories in accordance with how conserved they are (i.e., high, moderate, low, and non-conserved). In this study, we identified 828 conserved miRNAs (820 in gp1a and eight in gp1b) and 491 non-conserved miRNAs (71 in gp2a, 238 in gp2b, 20 in gp3, and 162 in gp4; Supplementary Figure 3 and Supplementary Table 2). Furthermore, 410 novel miRNAs were detected (i.e., not in a public soybean miRNA database).

### miRNAs May Regulate Pollen Development by Targeting Transcription Factors

In plants, miRNAs mediate gene expression at the post-transcriptional level by cleaving mRNAs at specific sites (Bartel, 2009; Shukla et al., 2011). The miRNA targets have been predicted using bioinformatics-based methods (Pla et al., 2018; Chen and Wang, 2020). Recently, a high-throughput method combining 5’ RACE and next-generation sequencing technology was developed to identify miRNA targets (Meng et al., 2010). In the present study, 1,617 genes were predicted to be targeted by 349 miRNAs in JLCMS9A and JLCMS9B. The predicted target genes encoded proteins that mediate a wide range of biological processes. Most of the miRNA targets were transcription factor genes (775 genes), including an auxin response factor, an AP2-like factor, a zinc knuckle (CCHC-type) family protein, a MYB domain family protein, a NAC domain-containing protein, a TCP transcription factor, an F-box family protein, and a basic helix–loop–helix DNA-binding superfamily protein. The target genes also encoded a splicing factor, a nucleosome assembly protein, and heat shock protein 70. Interestingly, a few targets identified in our degradome analysis were previously reported to be involved in plant reproductive development, including MYB proteins controlled by miR159 (Rhoades et al., 2002), AP2-like transcription factors targeted by miRNA172 (Aukerman and Sakai, 2003; Chen, 2004), and ARF6 or ARF8 regulated by miR167 (Wu et al., 2006). In this study, we predicted that the expression of the genes for 29 MYB domain proteins, 21 AP2-like proteins, and 44 ARF6 or ARF8 proteins is regulated by miR159, miR172, and miR167, respectively. Accordingly, miRNAs may regulate pollen development by targeting transcription factor genes. Our findings are consistent with those of a previous study on cotton (Yu et al., 2020).

### Abnormal Cell Wall Metabolism May Be an Important Factor Leading to Pollen Abortion in JLCMS9A

Pollen cell wall development is a crucial part of pollen production, and an abnormal pollen cell wall may be associated with male sterility in plants (Li et al., 2006; Ma et al., 2007; Wijeratne et al., 2007; Wei et al., 2013a; Hu et al., 2016; Xu et al., 2017; Zhou et al., 2017; Chen et al., 2018, 2019; Hamid et al., 2019; Nugent et al., 2019; Sutthinon et al., 2019; Mondol et al., 2020). Some genes controlling pollen development have been identified and cloned (Shi et al., 2011), including *DMD1* (Ren et al., 2020), *DPW2* (Xu et al., 2017), and *DPW3* (Mondol et al., 2020). In the current study, we identified 440 DEGs between JLCMS9A and JLCMS9B (Supplementary Table 4), with 428 and 12 of these genes expressed at lower and higher levels, respectively, in JLCMS9A than in JLCMS9B. The GO functional analysis assigned the 440 DEGs to 25 categories (10 biological processes, four cellular components, and 11 molecular functions; Figure 4A). Among the biological process categories, pectin catabolic process was the main functional term, followed by cell wall modification and catalytic activity. Regarding the cellular component categories, cell wall was the most significantly enriched functional term. Of the molecular function categories, pectinesterase activity was the main enriched GO term, followed by aspartyl esterase activity and enzyme inhibitor activity.

### miR169 May Regulate Pollen Development in Cytoplasmic Male Sterile Soybean

Earlier research confirmed that miR156, miR167, and miR399 contribute to pollen development in *A. thaliana* and a citrus species (Wu et al., 2006; Wang et al., 2009, 2020; Xing et al., 2010; Wei et al., 2012). To functionally characterize the differentially expressed miRNAs and miRNA target genes, we developed a regulatory network that included GO terms and KEGG pathways. The five GO terms for the regulatory network with 204 target genes and 55 miRNAs were sexual reproduction, anther development, meristem maintenance, meristem initiation, and nucleus (Figure 5). The five KEGG pathways in the regulatory network for 103 target genes and 42 miRNAs were ribosome, RNA degradation, RNA transport, spliceosome, and plant hormone signal transduction (Figure 6). The gma-miR169 family was involved in the regulation of the most miRNA target genes. Many studies proved that miR169 is a ubiquitous regulator of plant responses to various abiotic stresses (heat, cold, dehydration, and salt) and pathogens as well as developmental pathways (Liu et al., 2013; Ni et al., 2013; Potkar et al., 2013; Sorin et al., 2014; Xu et al., 2014, 2016; Luan et al., 2015; Hanemian et al., 2016; Du et al., 2017; Li et al., 2017; Serivichyaswat et al., 2017; Rao et al., 2020). However, to the best of our knowledge, there are no reports describing the involvement of miR169 in the male sterility of soybean or other plant species.

## Conclusion

In this study, we performed high-throughput sequencing and degradome analyses to identify miRNAs and their targets in a soybean CMS line (JLCMS9A) and its maintainer line (JLCMS9B). Additionally, DEGs during reproductive development were identified using RNA-seq data. The target genes that were revealed as differentially expressed between the CMS line and the maintainer line by an RNA-seq analysis were annotated with diverse functions related to biological processes, cellular components, and molecular functions, including transcriptional regulation, the nucleus, meristem maintenance, meristem initiation, cell differentiation, auxin-activated signaling, plant ovule development, and anther development. Finally, a network was built based on the interactions. Analyses of the miRNA, degradome, and transcriptome datasets generated in this study provided a comprehensive overview of the reproductive development of a CMS soybean line. The data presented herein represent useful information for soybean hybrid breeding. Furthermore, the study results indicate that miRNAs contribute to the soybean CMS regulatory network by modulating the expression of CMS-related genes.

## Materials and Methods

### Plant Materials and Growth Conditions

The RN–CMS soybean line JLCMS9A and its maintainer line JLCMS9B were used in this study. All plants were grown using a randomized block design (three replicates) at the Jilin Academy of Agricultural Sciences, China. More specifically, plants were cultivated in rows (5 m long and 65 cm wide), with 15 cm between plants. Mature flower buds were collected from 12 plants per genotype and stored at -80°C prior to the RNA-seq and sRNA-seq analyses, which were completed using three biological replicates per genotype.

### Small RNA Library Construction and Sequencing

Total RNA was extracted using TRK-1001 (LC Sciences, Houston, TX, United States) following the manufacturer’s instructions. The RNA quantity and purity were determined using the 2100 Bioanalyzer system and the RNA 6000 Nano LabChip Kit (Agilent Technologies, Santa Clara, CA, United States). High-quality RNA samples were those with an RNA integrity number greater than 7.0. Total RNA was ligated to the RNA 30 and RNA 50 adapters, then reverse transcribed and amplified by PCR to produce cDNA constructs of the sRNAs. The small cDNA fractions (22–30 nt long) were then isolated via 6% denaturing polyacrylamide gel electrophoresis. Finally, the cDNA constructs were purified, and the library was validated. We then performed single-end sequencing (50 bp) on an Illumina HiSeq 2500 system at LC-BIO (Hangzhou, China) following the vendor’s recommended protocol.

### Identification of Known and Potential Novel miRNAs

Raw reads were analyzed using ACGT101-miR (LC Sciences, Houston, TX, United States) to remove adapter dimers, junk reads, reads with low complexity, reads for common RNA families (rRNA, tRNA, snRNA, and snoRNA), and repeats. Unique sequences (18--25 nt long) were mapped to precursors in specific species in miRBase 21.0 on the basis of a BLAST search to identify known miRNAs and novel 3p- and 5p-derived miRNAs. Length variations at the 3’ and 5’ ends and one mismatch within the sequence were allowed during the alignment. The unique sequences mapped to the hairpin arm corresponding to a mature miRNA were identified as known miRNAs. The unique sequences mapped to the other hairpin arm were considered to be novel 5p- or 3p-derived miRNA candidates. The remaining sequences were mapped to precursors in other selected species in miRBase 21.0 on the basis of a BLAST search. The mapped pre-miRNAs were used as queries for a BLAST search of genomes from specific species to determine their genomic locations. The above two were designated as known miRNAs. The unmapped sequences served as queries for a BLAST search of specific genomes, and the hairpin RNA structures containing these sequences were predicted according to the 120-nt flanking sequences using the RNAfold program^2^. The following criteria were used for predicting secondary structures: (Virmani, 2012) number of nucleotides in one stem bulge ≤ 12 (Ivanov and Dymshits, 2007) number of base pairs in the stem region of the predicted hairpin ≥ 16 (Yuan, 1966) free energy (kCal/mol) cut-off ≤-15, (Bosacchi et al., 2015) hairpin length (upstream and downstream of stems and the terminal loop) ≥50 (Zhao et al., 2004) hairpin loop length ≤ 200 (Sun et al., 2001) number of nucleotides in one bulge in the mature region ≤ 4 (Banisch et al., 2012) number of biased errors in one bulge in the mature region ≤ 2 (Chambers and Shuai, 2009) number of biased bulges in the mature region ≤ 2 (Grant-Downton et al., 2009) number of errors in the mature region ≤ 4 (Shen et al., 2011) number of base pairs in the mature region of the predicted hairpin ≥ 12, and (Wang et al., 2009) proportion of the mature region in the stem ≥ 80%.

### Analysis of Differentially Expressed miRNAs

Differentially expressed miRNAs revealed by the normalized deep-sequencing read counts were analyzed by Student’s *t*-test. The following criteria were used to identify significantly up-regulated and down-regulated miRNAs: log_2_ expression level fold-change ≥ 1 and *P* < 0.05.

The expression of six selected miRNAs was assayed in JLCMS9A and JLCMS9B using Platinum SYBR Green-based q-RT-PCR (Invitrogen, United States) with analytikjena-qTOWER2.2 (Analytik Jena, Germany). The primers of six selected miRNAs and internal control gene (U6 snRNA) are available in Supplementary Table 7.

### Prediction of miRNA Target Genes

To predict the genes targeted by the most abundant miRNAs, computational target prediction algorithms (Target Finder) were used to identify miRNA binding sites. The predicted miRNA target genes were annotated with GO terms and assigned to KEGG pathways.

### Degradome Sequencing and Target Identification

Two degradome libraries were constructed as previously described (Ma et al., 2010). Polyadenylated RNAs were obtained and ligated to a 5p adapter, after which cDNA was generated by PCR. The cDNA was purified and then sequenced using an Illumina HiSeq 2500 system (LC Sciences, Hangzhou, China). By eliminating the low-quality data, the raw reads were obtained using Illumina Pipeline (version 1.5). After removing ADTs and reads shorter than 15 nt, the remaining reads were compared with the sequences in a cDNA library in the soybean genome database. The mapped reads were then aligned with the identified miRNAs using CleaveLand 3.0 (alignment score ≤ 4). Furthermore, on the basis of the number of degradome sequences and their abundance values, the miRNA targets were classified into five categories (0, 1, 2, 3, and 4) as previously described. To further elucidate their potential functions, the miRNA target genes were annotated using the GO and KEGG databases.

### Total RNA Extraction, cDNA Library Construction, and Ion Proton Deep Sequencing

Total RNA was extracted from each sample using TRIzol Reagent (Life Technologies, United States) according to the manufacturer’s protocol. The concentration of each sample was determined using the NanoDrop 2000 spectrophotometer (Thermo Scientific, United States), whereas the quality was assessed using the Agilent 2200 TapeStation system (Agilent). A sequencing library for each RNA sample was prepared using the Ion Total RNA-Seq Kit (version 2) according to the manufacturer’s protocol (Life Technologies). Briefly, polyadenylated mRNA was purified from 5 μg of total RNA using Dynabeads (Life Technologies). The mRNA was fragmented using RNase III and purified; after which, it was hybridized and ligated with an ion adapter. The RNA fragments were reverse transcribed and amplified to produce double-stranded cDNA, which was then purified using magnetic beads. After determining the molar concentration of each cDNA library, an emulsion PCR amplification was performed using the cDNA library as a template. Template-positive Ion PITM Ion Sphere^TM^ Particles were enriched and loaded onto the ion PITM chip for sequencing.

### Analysis of RNA-seq Data

Raw data (raw reads) in the FASTQ format were first processed using in-house Perl scripts. During this step, clean data (clean reads) were obtained by removing reads containing adapters or poly-N sequences as well as low-quality reads. Additionally, Q20 and Q30 values and the GC content of the clean data were calculated. All downstream analyses were completed using the high-quality clean data. The reference genome and gene model annotation files available online were downloaded^3^. A reference genome index was built using Bowtie (version 2.2.3; Langmead et al., 2009), after which paired-end clean reads were aligned to the reference genome using TopHat (version 2.0.12; Trapnell et al., 2009). The TopHat program was used for mapping because it can generate databases of splice junctions from the gene model annotation files, resulting in better mapping than that produced by other non-splice mapping tools. The HTSeq program (version 0.6.1) was used to determine the number of reads mapped to each gene (Anders et al., 2015). The fragments per kilobase of transcript per million base pairs sequenced (FPKM) value was then calculated for each gene based on the gene length and the number of reads mapped to the gene. Expression analyses involving the FPKM value are very common because they simultaneously consider the effects of sequencing depth and gene length on read counts. Pearson’s correlation coefficient among samples was used to evaluate the quality of the RNA-seq data.

### Analysis of Differential Expression

The differential expression between two conditions was analyzed using the DEGSeq R package (version 1.20.0; Anders and Huber, 2012). The *P* values were adjusted according to the Benjamini–Hochberg method. A corrected *P* value < 0.005 and a log_2_ (fold-change) of 1 were set as the threshold for identifying significant DEGs. The DEGs were functionally annotated with GO terms using the GOseq R package (Young et al., 2010), which corrects the gene length bias. Specifically, DEGs were annotated with GO terms on the basis of a corrected *P* value < 0.05.

The KEGG database comprises molecular information, including large-scale molecular datasets generated by genome sequencing and other high-throughput experimental technologies. It is useful for elucidating high-level functions and biological system activities (Kanehisa et al., 2008) in a cell, organism, and ecosystem^4^. We used the KOBAS program (Mao et al., 2005) to identify the enriched KEGG pathways among the DEGs.

## Data Availability Statement

The sequencing data has been deposited into the National Genomics Data Center (accession: CRA003993; link: https://bigd.big.ac.cn/search/?dbId=gsa&q=CRA003993&page=1).

## Author Contributions

CZ, FF, and LZ conceived and designed the study. CZ and FF performed the experiments and wrote the manuscript. JZ and BP collected the plant materials. FF and XD analyzed and modified the data. CL, HY, PW, and WZ provided advice and assistance. All authors have read and agreed to the published version of the manuscript.

## Conflict of Interest

The authors declare that the research was conducted in the absence of any commercial or financial relationships that could be construed as a potential conflict of interest.

## Footnotes

1 http://www.mirbase.org/

2 http://rna.tbi.univie.ac.at/cgi-bin/RNAfold.cgi

3 http://phytozome.jgi.doe.gov/pz/portal.html#!info?alias=Org_Gmax

4 http://www.genome.jp/kegg/

## Supplementary Material

Supplementary Figure 1 Distribution of known microRNAs (miRNAs) in JLCMS9A and JLCMS9B.

Supplementary Figure 2 Number of identified microRNAs (miRNAs) in each family.

Supplementary Figure 3 Number of identified conserved microRNAs (miRNAs).

Supplementary Figure 4 Detection of selected miRNAs expression in JLCMS9A and JLCMS9B using q-RT-PCR. U6 was chosen as an endogenous control. The reulsts were obtained from three biological replicates with three technical replicates and the error bars indicated the standard error of the mean.

Supplementary Table 1 Overview of small RNA sequencing reads from raw data to clean reads.

Supplementary Table 2 Summary of known and predicted microRNAs (miRNAs) in this study.

Supplementary Table 3 Distribution of microRNA family members.

Supplementary Table 4 Summary of the identified microRNAs (miRNAs) and their families in JLCMS9A and JLCMS9B flower buds.

Supplementary Table 5 Overview of the degradome sequences from JLCMS9A and JLCMS9B.

Supplementary Table 6 microRNA (miRNA) target genes in JLCMS9A and JLCMS9B.

Supplementary Table 7 The primers sequence of in this study.

## Abbreviations

CMS: cytoplasmic male sterility
miRNA: microRNA
sRNA: small RNA
DEG: differentially expressed gene
FDR: false discovery rate.
