# Supplementary figures and images for: MicroRNAs Involved in Regulatory Cytoplasmic Male Sterility by Analysis RNA-seq and Small RNA-seq in Soybean
Source: Front Genet. 2021 May 12;12:654146. doi: 10.3389/fgene.2021.654146 (PMC8153375; doi:10.3389/fgene.2021.654146)

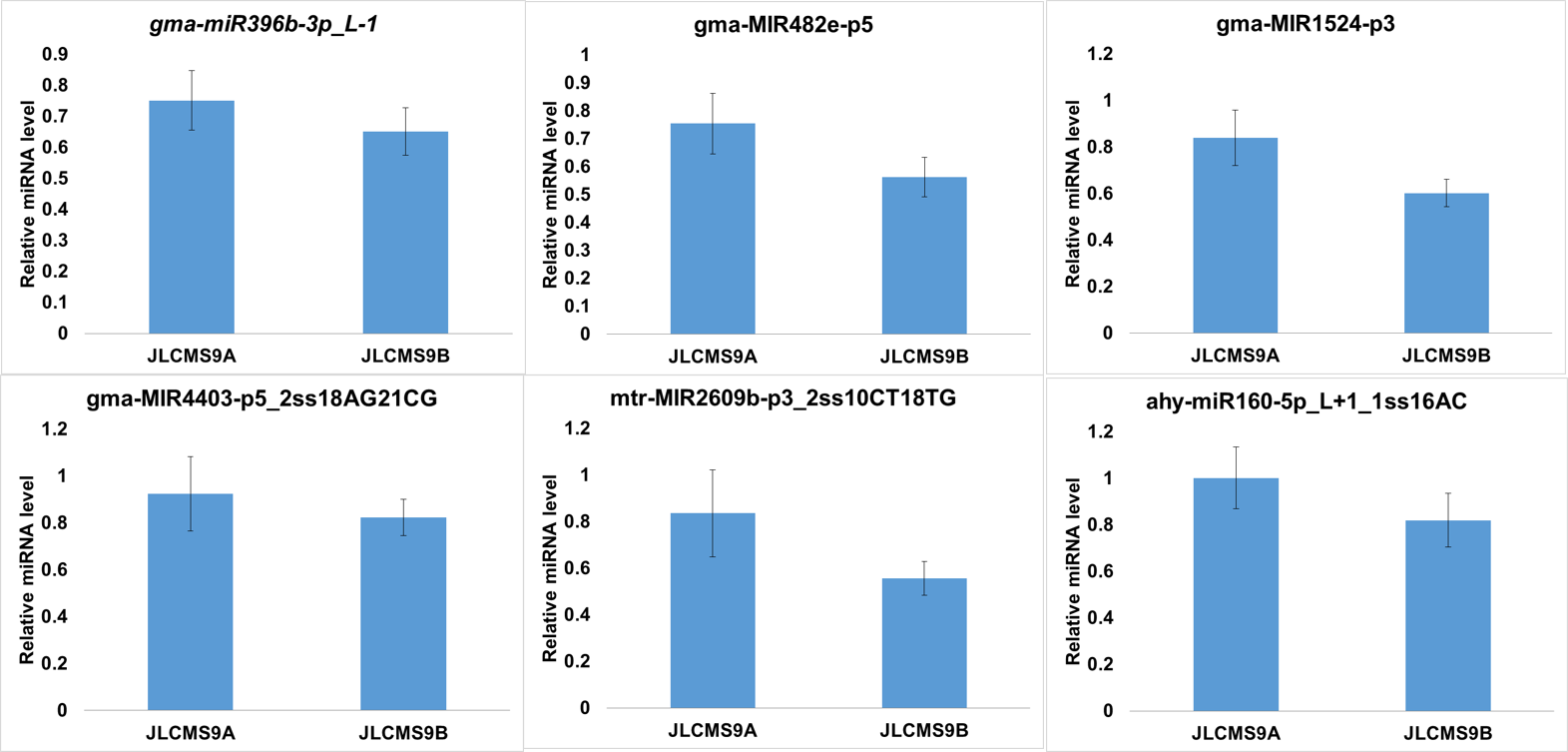

Supplement: Supplementary Figure 4 — Detection of selected miRNAs expression in JLCMS9A and JLCMS9B using q-RT-PCR. U6 was chosen as an endogenous control. The reulsts were obtained from three biological replicates with three technical replicates and the error bars indicated the standard error of the mean. [file Image_4.TIF]
